# Experimental demonstration of broadband solar absorption beyond the lambertian limit in certain thin silicon photonic crystals

**DOI:** 10.1038/s41598-020-68704-w

**Published:** 2020-07-16

**Authors:** Mei-Li Hsieh, Alex Kaiser, Sayak Bhattacharya, Sajeev John, Shawn-Yu Lin

**Affiliations:** 1 Department of Physics, Applied Physics, and Astronomy and Center-for-Future-Energy-System (CFES), Rensselaer Polytechnic Institute, 110 8th Street, Troy, NY 12180 USA; 2 Department of Photonics and the College of Photonics, National Chiao-Tung University, Hsinchu, Taiwan; 3 Department of Physics, University of Toronto, 60 St. George Street, Toronto, ON M5S 1A7 Canada; 4 Department of Electronics and Communication Engineering, Indraprastha Institute of Information Technology, New Delhi, 110020 India

**Keywords:** Energy science and technology, Materials science, Nanoscience and technology, Optics and photonics

## Abstract

The tantalizing possibility of 31% solar-to-electric power conversion efficiency in thin film crystalline silicon solar cell architectures relies essentially on solar absorption well beyond the Lambertian light trapping limit (Bhattacharya and John in Nat Sci Rep 9:12482, 2019). Up to now, no solar cell architecture has exhibited above-Lambertian solar absorption, integrated over the broad solar spectrum. In this work, we experimentally demonstrate two types of photonic crystal (PhC) solar cells architectures that exceed Lambertian light absorption, integrated over the entire 300–1,200 nm wavelength band. These measurements confirm theoretically predicted wave-interference-based optical resonances associated with long lifetime, slow-light modes and parallel-to-interface refraction. These phenomena are beyond the realm of ray optics. Using two types of 10-μm thick PhC’s, first an Inverted Pyramid PhC with lattice constant a = 2,500 nm and second a Teepee PhC with a = 1,200 nm, we observe solar absorption well beyond the Lambertian limit over λ = 950–1,200 nm. Our absorption measurements correspond to the maximum-achievable-photocurrent-density (MAPD), under AM1.5G illumination at 4-degree incident angle, 41.29 and 41.52 mA/cm^2^ for the Inverted Pyramid and Teepee PhC, respectively, in agreement with wave-optics, numerical simulations. Both of these values exceed the MAPD (= 39.63 mA/cm^2^) corresponding to the Lambertian limit for a 10-μm thick silicon for solar absorption over the 300–1,200 nm band.

## Introduction

The efficiency and cost of photovoltaics has steadily improved in recent years in the effort to create a competitive renewable energy resource. Silicon solar cells have been the dominant driving force in photovoltaics due to the abundance and environmentally friendly nature of silicon. The maximum possible power conversion efficiency of a single junction, crystalline silicon (*c*-Si) solar cell under one sun illumination at room temperature is 32.33%^2^. The highest efficiency real-world n-type silicon solar cell to date, by Kaneka Corp^3,4^, exhibits 26.7% conversion efficiency, followed closely by the p-type silicon solar cell, by the Institute for Solar Energy Research Hamelin (ISFH), Germany with 26.1% efficiency^5,6^. An analysis of the Kaneka, 165 μm thick, *c*-Si cell shows that in the absence of any extrinsic loss mechanism, the limiting efficiency of such a cell is 29.1%^3^. The competing factors responsible for this limit of the conversion efficiency are ray-optics light trapping^7,8^ and intrinsic loss due to Auger charge-carrier recombination. Essentially, the thicker the cell, the more light is absorbed. However, this is accompanied by increased bulk non-radiative recombination loss of charge carriers. In the case of ideal Lambertian light-trapping and a state-of-the-art Auger recombination model^9^, the optimal silicon thickness is reduced to 110 μm and a theoretical limit to conversion efficiency (assuming no surface recombination losses) is increased to 29.43%^8^. In traditional ray-optics based light trapping structures, the Lambertian limit is not achieved, the optimum solar cell thickness is much greater than 110 μm and the power conversion efficiency is not expected to go beyond 28%.

The wave nature of light offers an alternative paradigm for solar energy capture in silicon. This is evident in certain sub-wavelength scale waveguides^10–13^ and photonic crystals^14,15^. While traditional 2D photonic crystals guide light in the 2D plane^16^, our new type of 2D photonic crystal deflects sunlight from the z-direction and couples it into the x–y plane^17,18^. In contrast to 165 μm-thick Kaneka cell and 110 μm-thick Lambertian cell, the PhC cells are 10–15 μm thick and have conversion efficiencies exceeding 30%^1,19,20^. The key mechanisms enabling the 30% cell using just 10–15 μm thick silicon are the existence of slow-light resonances and parallel-to-interface refraction (PIR)^21^. Particularly, using an inverted nano-pyramid thin silicon photonic crystal, light trapping toward the Lambertian limit has been reported^22,23^. Additionally, in a separate metal-oxide PhC system, PIR effect was experimentally demonstrated to yield two order-of-magnitude enhancement of optical absorption^24^. Light waves in PhCs exhibit behavior beyond the realm of ray-optics and have the potential to bridge the gap between the thermodynamic efficiency limit and ray-optics based limits.

In this paper, we constructed square-lattice PhC solar cell structures that support PIR modes and exhibit solar absorption well beyond the Lambertian limit. We realized two types of 10-μm thick simple-cubic PhC, the inverted pyramid PhC with lattice constant a = 2,500 nm and the Teepee PhC with a = 1,200 nm. Despite the fact that these structures are not fully optimized^19,20^ for solar light trapping, we observe that both PhC structures exhibit solar absorption well beyond the Lambertian limit in the weakly absorbing near-infrared regime, λ = 950–1,200 nm. Furthermore, we found the maximum-achievable-photocurrent-density (MAPD) under AM1.5G illumination at 4-degree incident angle to be 41.29 and 41.52 mA/cm^2^ for the inverted pyramid and the Teepee PhC, respectively. These values exceed the MAPD (= 39.63 mA/cm^2^) corresponding to the Lambertian limit for a 10-μm thick silicon.

## Sample design and fabrication

**Figure 1:**
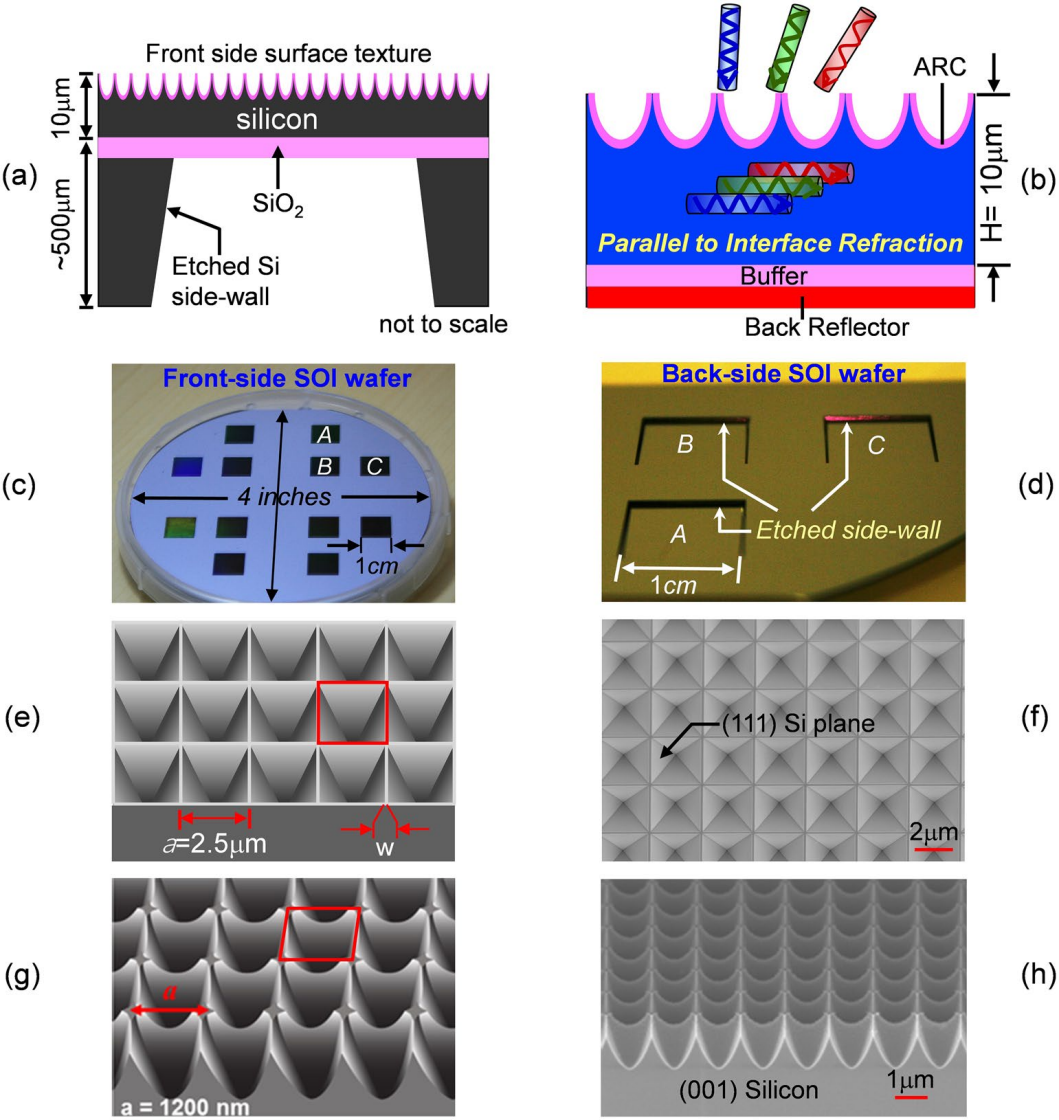
(**a**) A schematic cross section view of the SOI (silicon-on-insulator) sample structure. Its front surface has a periodic surface texture and its back surface etched, leaving behind a 10 μm thick crystalline silicon; (**b**) a magnified schematic of the sample coated with a front ARC (antireflection) layer and a back-side SiO_2_ buffer and a back reflector; (**c**) a photo of the front side of a 4-inch SOI wafer, having a periodic surface texture; (**d**) a photo of the back side of the SOI wafer, showing the etched silicon side-wall. (**e**) A schematic of the Inverted Pyramid Photonic-Crystal (PhC) with a lattice constant *a* = 2,500 nm; (**f**) a SEM (scanning electron micrograph) image of the fabricated Inverted Pyramid structure. A wet etch method is used to etch and expose the silicon (111) surfaces, as indicated by the black arrow. (**g**) A schematic of the Teepee PhC with a lattice constant *a* = 1,200 nm. (**h**) A SEM image of the fabricated Teepee PhC structure. The etched surface follows a Gaussian-like graded index profile.

Our devices were fabricated on SOI (silicon-on-insulator) wafers using standard micro-electronic lithographic and etching processes. The Inverted Pyramid and Teepee PhC were fabricated at Australia’s Melbourne Centre for Nanofabrication Facility (MCN)^25^ and at Cornell’s Center for Nano-Fabrication (CNF)^26^, respectively. Figure 1a shows a schematic design of the thin PhC silicon solar cell architecture. The dimension shown is not to scale. The buried SiO_2_ layer is 250 nm thick and the handling silicon substrate is 500 μm thick. The front-side surface texture consists of a two-dimensional PhC with square lattice symmetry. Each cell is one square centimeter in area. It is defined using lithography and isolated from each other by etching away the surrounding silicon. Figure 1b shows a magnified schematic of the sample coated with a front-side antireflection (ARC) layer and a back-side metallic reflector. In this PhC design, the incident sunlight at long wavelengths is refracted into the thin silicon layer and propagates through resonant modes nearly parallel-to-the-interface. Figure 1c shows a photo of the front-side of the fabricated SOI wafer. Cell-A and –B have an identical PhC structure. Cell-C is the reference cell and has no surface texture. The backside wafer is produced using wet chemical etching in a KOH solution. Figure 1d shows a photo of the etched silicon side-wall. The etch sidewall is well-defined, leaving behind a 10-μm thick silicon and 250 nm buried SiO_2_ layers.

Both the Inverted Pyramid and Teepee PhCs were theoretically optimized^19,20^ and the fabricated structures are not far from the optimal geometries. Figure 1e,f shows a schematic design and SEM (scanning electron micrograph) image of the IP structure, respectively. The IP structure has a square-lattice symmetry as indicated by the red square and a lattice constant a = 2.5 μm. It is produced by patterning the front-side of the wafer and, subsequently, etching the pyramid shape mesa using KOH chemical solution. The silicon etch is orientation selective and stops at the (111) crystal plane, leaving behind a well-defined surface facet with a tilt angle of ~ 54° (indicated by the black arrow). The resulting PhC has an etched height h = 1.7 μm and an aspect ratio h/(a/2) = 1.36. The mesa width between adjacent pyramids is 25–100 nm. Figure 1g,h shows a schematic design and SEM image of the fabricated Teepee PhC structure, respectively. It also possesses square-lattice symmetry, but has a curved surface profile like a Teepee-shape. This particular surface profile is achieved by first patterning the front-side of the wafer, followed by a one-step RIE etching process in crystalline silicon. The bottom of the trenches are sharp, with sidewall angles of ~ 70°. The resulting PhC has a lattice constant a = 1,200 nm, an etched height h = 1.4 μm and an aspect ratio h/(a/2) ~ 2.3. This is not far from the theoretical optimal geometry^19^ with a lattice constant a = 1,000 nm. Furthermore, the etched profile may be approximated by the Gaussian and/or parabolic function that leads to superior broadband and wide angle antireflection^27–29^. Accordingly, the Teepee PhC is also referred to as the Parabolic Pore PhC^19^. Due to the dry etch process, some silicon surface roughness is evident. While this does not affect optical absorption, it could lead to additional charge carrier recombination centers compared to a smooth surface. To partially compensate for surface damage, a thin SiO2 coating (t ~ 60 nm) by high temperature oxidation and annealing was introduced, providing some surface passivation as well as improved antireflection property.

## Results and discussion

**Figure 2:**
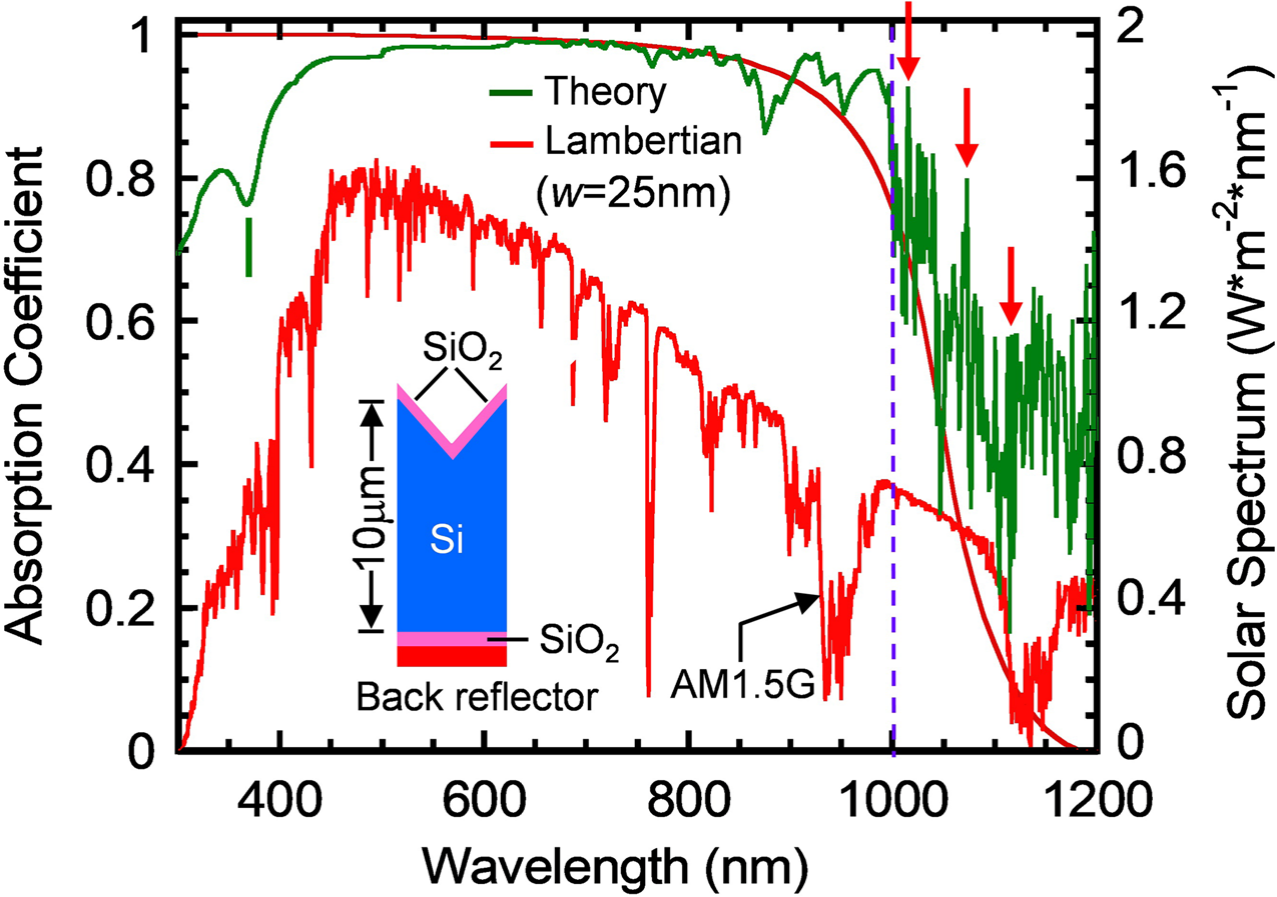
A computed absorption spectra of a 10 μm-thick silicon Inverted Pyramid (IP) structure at normal incidence. The structure has lattice constant *a* = 2,500 nm, a single layer ARC (100 nm), a back-side SiO2 buffer layer (100 nm) and a Al back-reflector. The Lambertian absorption (the red curve) and AM1.5G solar radiation spectra are also shown as references. The IP’s absorption approaches the Lambertian curve over the λ ~ 550–900 nm region, slightly exceeds the Lambertian limit over the λ ~ 900–1,000 nm and far exceeds the Lambertian limit over the λ = 1,000–1,200 nm region. The red arrows indicate strong resonance peaks at λ = 1,016, 1,070 and 1,110 nm.

Figure 2 shows the computed absorption spectra, at normal incidence, of a 10-μm thick Inverted Pyramid PhC structure (the green curve). The structures have lattice constant *a* = 2,500 nm, a single, 100 nm, SiO_2_ layer of ARC, a 100 nm back-side SiO_2_ buffer layer and an Aluminum back-reflector. This is not far from the optimized structure with a dual layer ARC of 145 nm thickness^20^. The Lambertian absorption spectrum (the red curve) and AM1.5G solar spectrum are also shown as references. The computed IP PhC’s absorption approaches the Lambertian curve in the λ ~ 550–900 nm range, slightly exceeds the Lambertian limit in the λ ~ 900–1,000 nm range and far exceeds the Lambertian limit in the λ = 1,000–1,200 nm range. In the λ = 1,000–1,200 nm range, multiple resonant peaks occur and three of which are indicated by red vertical arrows at λ = 1,016, 1,070 and 1,110 nm. These strong absorption peaks originate from higher order PhC modes that are long lifetime, slow-light resonance, parallel-to-the-interface refraction^1^. The parallel flow of electromagnetic waves increases the optical path-length by an order-of-magnitude within the 10*-*μm thick silicon and, hence, dramatically increases optical absorption in this otherwise weakly absorbing near-infrared region. The absorption dip for λ < 400 nm is due to strong specular reflection of sunlight as the real part of refractive index of silicon exhibits a sharp peak in this range [see Fig. 1 of A. Deinega and S. John, Optics Letter 37, no.1, p. 112–114 (2012)].

Following the computational analysis, we perform a series of absorption measurements of the IP and Teepee PhCs. Total absorption measurements were performed using an integrating sphere (Labsphere) with an unpolarized xenon light source, and an Ocean Optics HR2000 + spectrometer was used for data collection. The sample was inserted in the middle of the sphere and the absorption (A) was measured. For angular dependence measurements, the sample holder was rotated accordingly with respect to the incident light beam. For each sample, we performed measurement for a range of angles at θ = 4, 10, 20, 30, 40, 50 and 60°. The absorption at normal incidence (0°) was neglected because the specular reflection from the sample surface would escape from the integrating sphere. Instead, the smallest light incident angle we used for our experiment is θ = 4°. For the θ = 4° testing, our light source has a beam diameter of ~ 10 mm so that it illuminates the entire test sample piece of 10 × 10mm^2^. For all other incident angles, the beam diameter is reduced to ~ 5 mm to accommodate tilt angle testing of a finite size sample. For absorption measurements in the near IR wavelength (λ = 900–1,200 nm), Near Infrared Spectrometer (Ocean Optics NIRQuest512-2.5) was used for data collection with a high-quality thermoelectric-cooled InGaAs linear array (Hamamatsu G9208-512 W) and an unpolarized tungsten-halogen light source.

**Figure 3:**
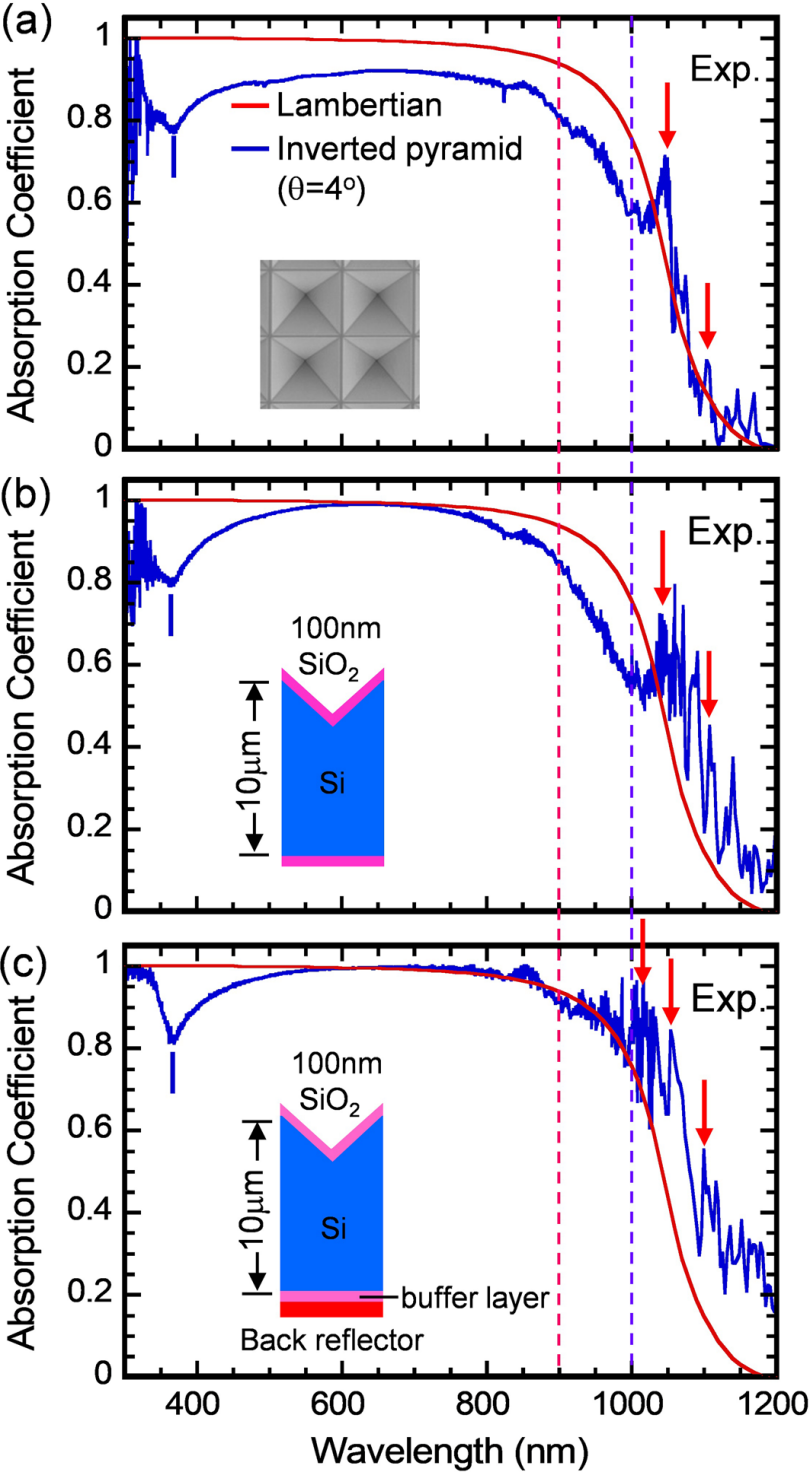
Experimentally measured absorption spectra of a 10-μm thick silicon IP cell, having lattice constant *a* = 2,500 nm. (**a**) Absorption spectrum measured for an IP structure without either an ARC layer, a buffer layer, or the back-reflector (the blue curve). The red curve corresponds to the Lambertian absorption limit. Over λ = 1,000–1,200 nm region, multiple high resonant absorption peaks are observed (indicated as the red arrows). (**b**) Spectrum measured for the same structure, but only coated with a ~ 100 nm thick SiO_2_ ARC. There is no back reflector. (**c**) Spectrum measured for the same structure coated with a front SiO2 ARC, a back-side ~ 100 nm SiO2 buffer layer and a back reflector. Over λ = 1,000–1,200 nm region, the multiple resonant peaks become more pronounced and far exceed the Lambertian absorption limit.

Figure 3a shows spectrum measured for an Inverted Pyramid sample of 10-μm thick (the blue curve) over the ultraviolet, visible and near-infrared wavelengths (λ = 300–1,200 nm). This sample has lattice constant *a* = 2,500 nm, a mesa-width of w = 100 ± 10 nm, and has no ARC or back reflector. The red curve is the Lambertian absorption limit for a 10-μm thick silicon cell. For λ = 300–1,000 nm, the measured absorption is below the Lambertian limit. For λ = 1,000–1,200 nm, multiple high resonant absorption peaks are observed (indicated as the red arrows) which slightly exceed the Lambertian limit. Figure 3b shows the spectrum measured for the same sample, but coated with a ~ 100 nm thick SiO_2_ ARC, a 100 nm SiO_2_ buffer layer, and no Al-back-reflector. The ARC coating is to reduce optical reflection loss at the air-PhC interface. Indeed, the absorption at λ = 550–750 nm is increased and approaches the Lambertian curve. Furthermore, the resonant absorption peaks at λ = 1,000–1,200 nm become more pronounced and exceeds the Lambertian limit. Finally, Fig. 3c shows the spectrum measured for the same sample coated with a front SiO_2_ ARC, a back-side ~ 100 nm SiO_2_ buffer layer and an Aluminum back reflector. The back reflector is to recycle the otherwise transmitted light and increase the absorption further. In this case, the absorption at λ = 550–900 nm approaches the Lambertian limit. Moreover, for the λ = 1,000–1,200 nm spectral range, the IP PhC’s absorption exceeds the Lambertian limit by orders of magnitude. The observed multiple resonant peaks at λ = 1,015, 1,055 and 1,100 nm agree with the predicted ones within Δλ = 15 nm. The slight discrepancy is likely due to a small amount of structure disorder across the fabricated sample surface. However, the average absorption over the band of sharp resonances in λ = 1,000–1,200 nm shows good agreement between theory and experiment. The MAPD over 300–1,200 nm range for cases Fig. 3a–c increases from 35.81, 38.56 to 41.29 mA/cm^2^, respectively. It is expected that with further optimization of the mesa width and a dual layer SiN and SiO_2_ ARC, this absorption can be enhanced to yield nearly an additional 2 mA/cm^2^ in the MAPD over the 300–1,200 nm range^1,20^.

**Figure 4:**
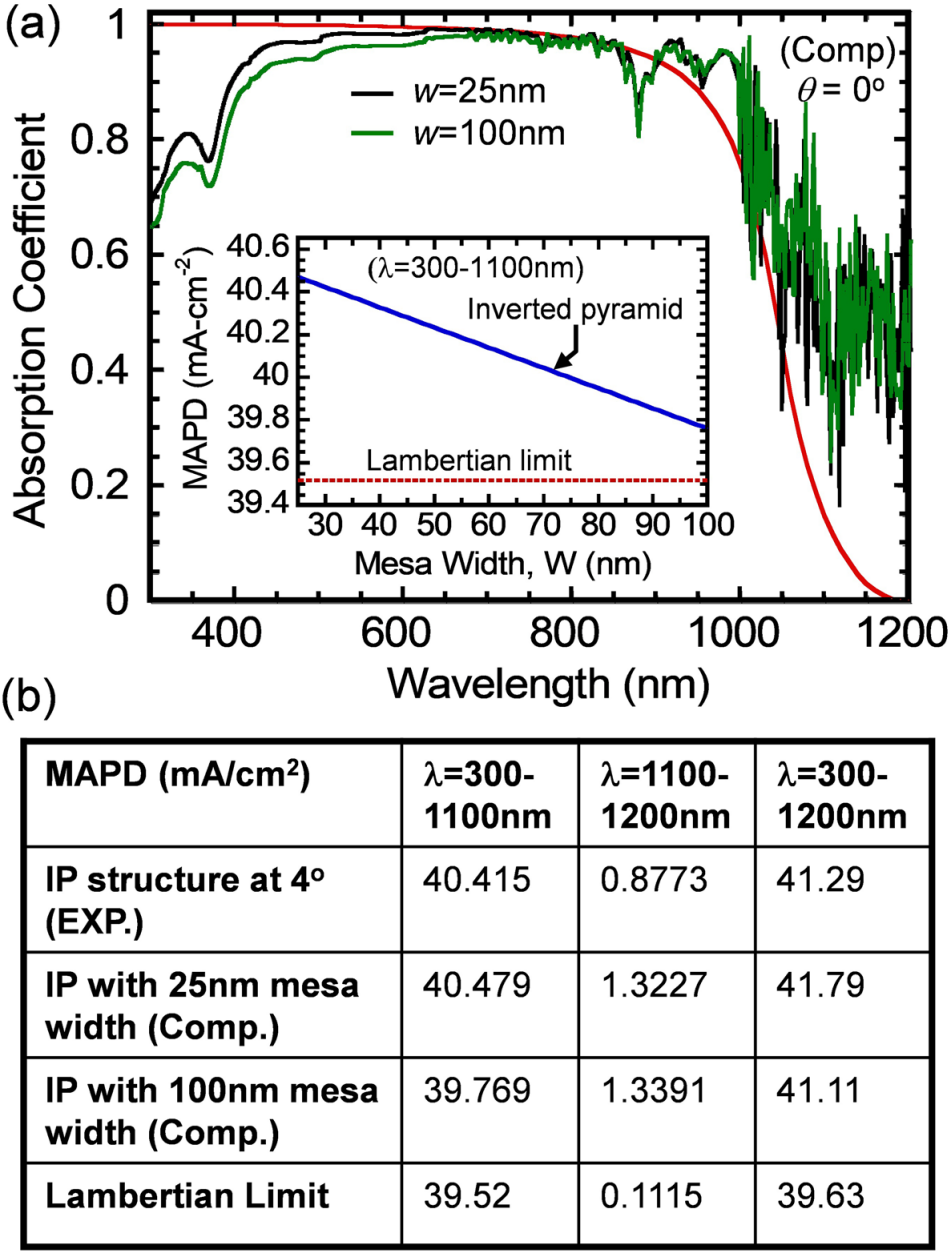
(**a**) Absorption spectra of a 10-μm thick IP cell structure computed for two different mesa-width *w* = 25 and 100 nm, respectively. The IP structure is coated with a single layer SiO_2_ ARC (100 nm), a buffer layer (100 nm), and an Al-back-reflector. The Lambertian absorption is also shown as the red curve. The inset shows a summary of MAPD over λ = 300–1,100 nm as a function of mesa-width *w*. (**b**) A summary of MAPD values over λ = 300–1,100, 1,100–1,200 nm and 300–1,200 nm regions for the 10-μm thick silicon IP cell structure. The MAPD corresponding to the Lambertian limit was also shown. The total MAPD over 300–1,200 nm range for the *w* = 25 nm and 100 nm IP PhC structure is 41.79 and 41.11 mA/cm^2^, respectively.

The final optimization of the IP cell structure is its mesa-width *w,* which is indicated in the SEM image in Fig. 1e. Figure 4a shows absorption spectra of 10-μm thick IP structures computed for two different mesa-width *w* = 25 nm (the black curve) and 100 nm (the green curve). The IP PhC structure is coated with a single layer SiO_2_ ARC (100 nm), a SiO_2_ buffer layer (100 nm) and a Al-back-reflector. The Lambertian absorption is also shown as a reference (the red curve). For the *w* = 25 nm cell structure, its absorption in the λ = 300–700 nm range is slightly higher due to a narrower mesa-width, resulting in less optical reflection loss. The inset of Fig. 4a shows a plot of the partial MAPD (over the 300 nm to 1,100 nm range) versus mesa-width *w* = 25–100 nm. MAPD of the cell structure under AM1.5*G* illumination is given by: $J_{MADP} = \int\nolimits_{{\lambda_{\min } }}^{{\lambda_{\max } }} {\frac{e\lambda }{{hc}}} I\left( \lambda \right)A\left( \lambda \right)d\lambda$. Here, I(λ) is the intensity of the AM1.5G solar radiation spectrum. We assume that each absorbed photon creates a single electron–hole pair. The short-circuit current (*J*_*sc*_) of an ideal cell, without surface and bulk recombination losses, coincides with *J*_*MAPD*_. The MAPD corresponding to the Lambertian limit for 10 μm-thick silicon cell is 39.52 mA/cm^2^ in the 300–1,100 nm range. For the 300–1,100 nm spectral range, MAPD corresponding to the 10-μm thick IP structure is increased from 39.769 to 40.479 mA/cm^2^ as *w* is decreased from 100 to 25 nm. By implementing an optimized dual layer SiN and SiO_2_ ARC, MAPD for the 10-μm thick PhC can be further increased to 42.5 mA/cm^2^^20^. Figure 4b shows a summary of the experimental and computed MAPD values obtained from the IP cell structure over λ = 300–1,100 nm, 1,100–1,200 nm (sub-gap region) and 300–1,200 nm region. The MAPD corresponding to the Lambertian limit for 10-μm thick silicon is also shown. Over λ = 300–1,100 nm region, MAPD values for the IP cells slightly exceed the Lambertian limit of 39.52 mA/cm^2^. Over the sub-gap region, λ = 1,100–1,200 nm, MAPD for the IP cells far exceed the Lambertian limit of 0.1115 mA/cm^2^. Finally, for the entire λ = 300–1,200 nm region, the measured MAPD corresponding to the 10-μm thick IP PhC is 41.29 mA/cm^2^ which exceeds the Lambertian limit of 39.63 mA/cm^2^ by 1.66 mA/cm^2^. For the specific structure fabricated, there is good overall agreement between theory and experiment. This agreement provides credence to theoretical prediction that with further optimization of the ARC and mesa structure, the MAPD could reach as high as 43.59 mA/cm^2^ over the 300–1,200 nm band^1^.

**Figure 5:**
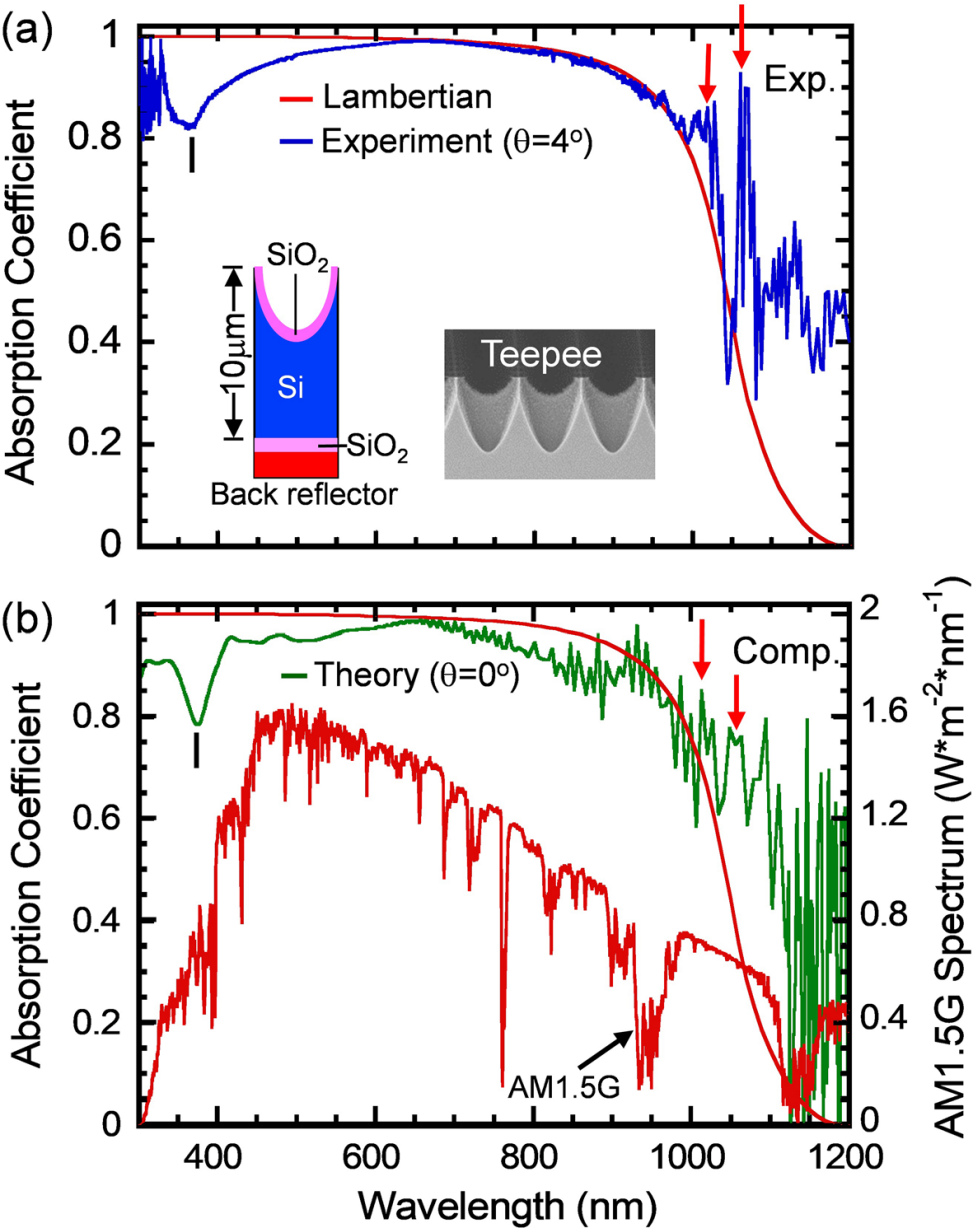
The (**a**) measured and (**b**) computed absorption spectra of the Teepee cell structure, having lattice constant *a* = 1,200 nm. Both structures are coated with a single layer ARC (60 nm), a back-side SiO_2_ buffer layer (75 nm) and Ag back-reflector. Both spectra agree with each other. Particularly, they both approach the Lambertian limit over λ ~ 550–1,000 nm region and exceeds the Lambertian limit over λ ~ 1,000–1,200 nm region. The predicted multiple resonant peaks at λ = 1,028 and 1,062 nm were also observed in the experiment (the red arrows).

While the Teepee and Inverted Pyramid PhCs possess the same square-lattice symmetry, the Teepee PhC cell structure has a deeper and more curved trench in its surface profile. This feature has been shown to yield a better broad bandwidth and wide angle absorption performance^18,29^. Figure 5a,b shows the measured and computed absorption spectra of the 10-μm thick, fabricated Teepee PhC structure with lattice constant *a* = 1,200 nm. The inset shows that the cell structure has a single layer ARC (60 nm), a back-side SiO_2_ buffer layer (75 nm) and Ag back-reflector (200 nm). It should be noted that both spectra agree with each other. They both exhibit a slight dip of A ~ 83% at λ ~ 375 nm, approach the Lambertian limit in the λ ~ 600–1000 nm range and exceed the Lambertian limit in the λ ~ 1,000–1,200 nm range. In the 700–900 nm range, the measured absorption spectrum is smoother than the computed one and also shows a slightly stronger absorption. The smear out of the computed resonance peaks in this range may be caused by local randomness in the fabricated structure, which in turn reduces the surface reflection and increases the Teepee structure’s absorption accordingly^30,31^. Finally, over 950–1,200 nm, the computed and measured spectra show multiple resonant peaks that agree with each other. For example, the absorption peaks at λ = 1,028 nm and 1,062 nm agree with each other and are indicated as red arrows. In contrast to Lambertian cell and planar cell structures, higher solar absorption in the 950–1,200 nm spectral range due to multiple resonant absorption peaks is a signature of photonic crystal light-trapping.

**Figure 6:**
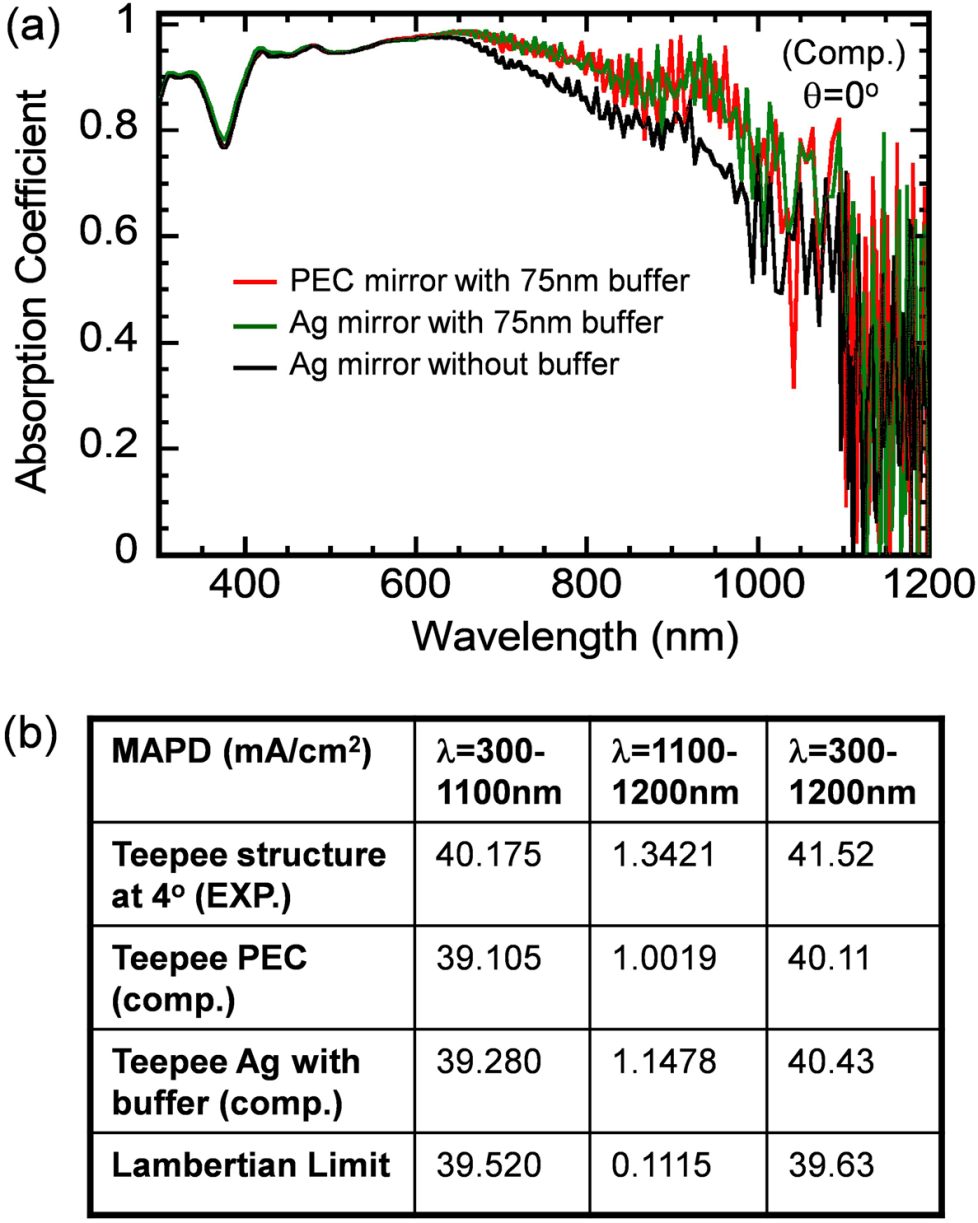
(**a**) Absorption spectra of a 10-μm thick silicon Teepee cell structure computed for three different back reflectors, i.e. the perfect electric conductor (PEC), Ag mirror with 75 nm SiO2 buffer and Ag mirror without buffer, respectively. The top SiO_2_ ARC is a 60 nm thick conformal ARC. (**b**) A summary of MAPD values for the 10-μm thick silicon Teepee cell structure with a single layer SiO_2_ ARC over λ = 300–1,100, 1,100–1,200 and 300–1,200 nm regions. In the presence of the buffer layer (75 nm), the parasitic absorption in Ag is 0.18 and 0.14 mA/cm^2^ over 300–1,100 nm and 1,100–1,200 nm, respectively.

We comment that while the use of a metallic back reflector can increase optical absorption of a thin film structure, it also can contribute to parasitic absorption. Further FDTD (finite difference time domain) calculations were performed to estimate such parasitic absorption losses in our cell structure. Figure 6a shows absorption spectra of a 10-μm thick Teepee structure computed for three different back reflectors, i.e. the perfectly electric conductor PEC (the red curve), Ag-mirror with 75 nm SiO_2_ buffer (the green curve) and also Ag-mirror without buffer (the black curve). The top SiO_2_ ARC is a 60 nm thick, conformal ARC. The use of either PEC or Ag-mirror with buffer leads to very similar absorption spectra over the entire spectral range of 300–1,200 nm. However, the use of the Ag-mirror without buffer reduces the absorption significantly especially over λ = 650–1,100 nm range. Figure 6b summarizes MAPD corresponding to the computed and measured absorption spectra of the 10-μm thick optimized Teepee cell structure. In the case of 75 nm buffer + 100 nm PEC mirror, MAPD over 300–1,200 nm range is 40.11 mA/cm^2^. In the case of 75 nm buffer + 100 nm Ag-mirror, MAPD over 300–1,200 nm range is 40.43 mA/cm^2^. Thus, in the presence of the buffer layer the parasitic absorption in Ag over 300–1,200 nm range is 0.32 mA/cm^2^. Note that, in the case of 75 nm buffer and 100 nm Ag mirror, MAPD over 300–1,100 nm range corresponding to the experimental and computational data is 40.175 and 39.280 mA/cm^2^, respectively. MAPD corresponding to the experimental data is higher than the computational one by 0.895 mA/cm^2^. This difference is due to the higher absorption measured in the 700–900 nm range, as explained in the previous paragraph. Finally, we note that MAPD over λ = 300–1,200 nm range for the 10-μm thick Teepee PhC structure-Exp. is 41.52 mA/cm^2^, which exceeds the Lambertian limit of 39.63 mA/cm^2^ by 1.89 mA/cm^2^. This is clearly beyond any effects that could arise from parasitic absorption in the back-reflector.

## Computational results

**Figure 7:**
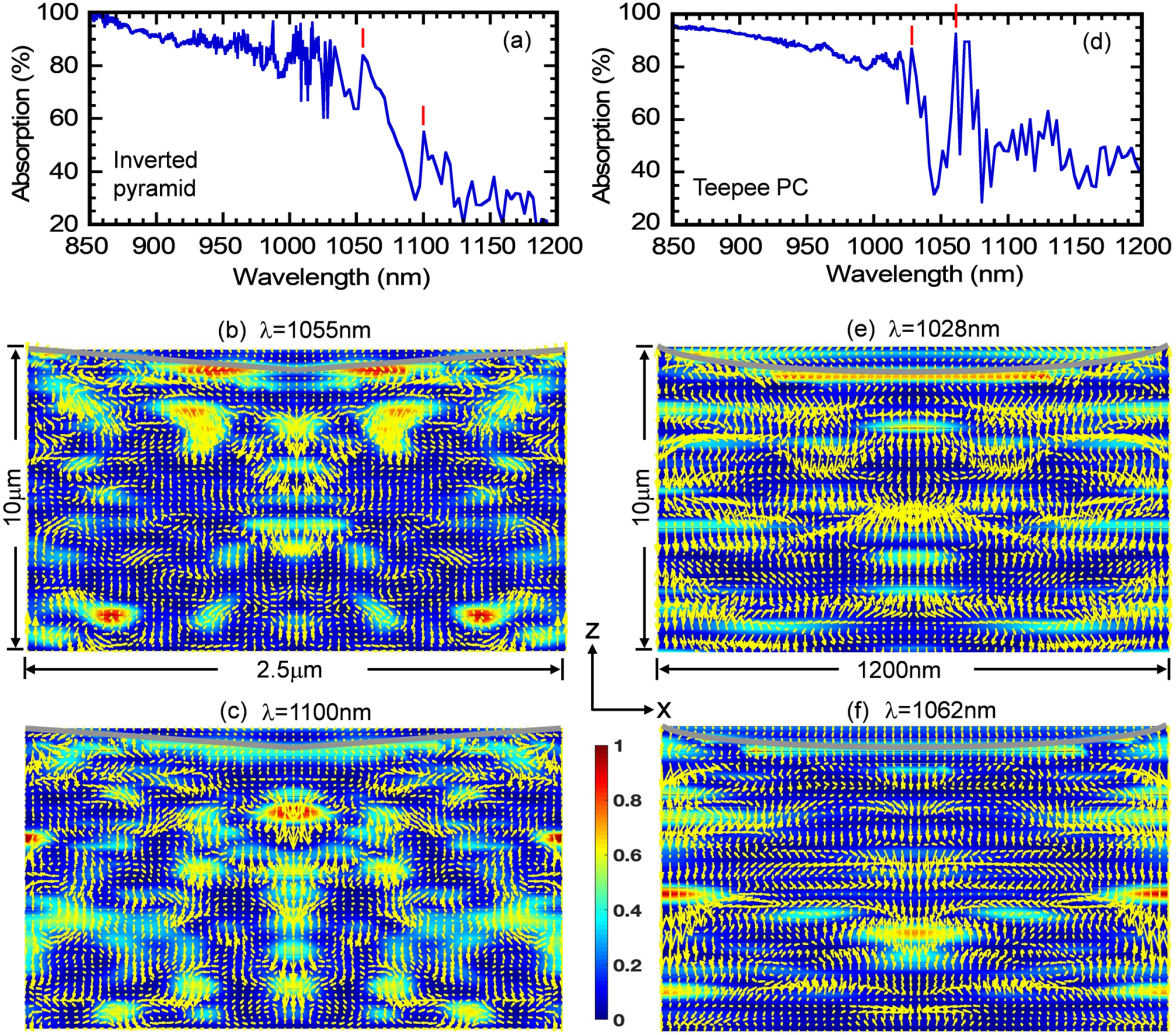
Vortex-like modes and Parallel-to-Interface refraction (PIR) in square-lattice photonic crystal samples. (**a**) A magnified absorption spectrum of the silicon IP photonic crystal sample over the 850–1,200 nm wavelength range. The red vertical bars correspond to the resonances at λ = 1,055 and 1,100 nm. (**b**) and (**c**) Yellow arrows show the in-plane Poynting vector flow in the Z–X planes of the IP PhC crystal. Such long life modes are responsible for the high absorption even in the 1,000–1,200 nm weak absorption wavelength range. (**d**) A magnified absorption spectrum of the Teepee photonic crystal sample over the 850–1,200 nm wavelength range. The red vertical bars correspond to the resonances at λ = 1,028 and 1,062 nm. (**e**) and (**f**) Yellow arrows show the in-plane Poynting vector flow in the Z–X planes of the Teepee PC.

These multiple peaks in the absorption spectra over λ = 1,000–1,200 nm originate from purely wave-interference effects, absent in Lambertian light-trapping. To illustrate this point, we show a magnified view of the absorption spectrum of the 10 μm-thick, optimized Inverted Pyramid PhC cell over the 850–1,200 nm wavelength range in Fig. 7a. The red vertical lines correspond to resonant absorption peaks located at λ = 1,055 and 1,100 nm. Figure 7b, c show the in-plane Poynting vector plots over the central xz-slice of the Inverted Pyramid PhC unit cell at these resonances. The Poynting vectors are superposed on the color map that represents $\left| {\text{E}} \right|^{2}$ (normalized by maximum of $\left| {\text{E}} \right|^{2}$). The grey lines in these plots denote the outline of the top of the silicon layer. The energy flow-pattern reveals multiple regions with vortex-like flow and parallel to interface flow of light at these resonances leading to very long dwell-time of photons in the solar cell. On the other hand, Lambertian light trapping assumes that the distribution rays in the cell obeys a probability distribution *f*(θ) = 1/π (cosθ), where θ is the angle that a ray within the cell makes with the cell-surface normal. According to this distribution, propagation of energy near θ = 90° (i.e. parallel to the interface) is insignificant. However, direct solutions of Maxwell’s equations show that a significant amount of energy flows close to θ = 90° due to wave-interference based light-trapping in our PhC structure. Similar computations were also performed for our Teepee PhC structure. Figure 7d show a magnified view of the absorption spectrum of the 10 μm-thick, optimized Teepee PhC cell over the 850–1,200 nm wavelength range. The red vertical lines correspond to resonant absorption peaks located at λ = 1,028 and 1,062 nm. Figure 7e, f show the in-plane Poynting vector plots over the central *xz*-slice of the Teepee PhC unit cell at these resonances. Again, the energy flow-pattern reveals multiple regions with vortex-like flow and parallel to interface flow of light at these resonances.

## Angular dependence of absorption spectra and MAPD

**Figure 8:**
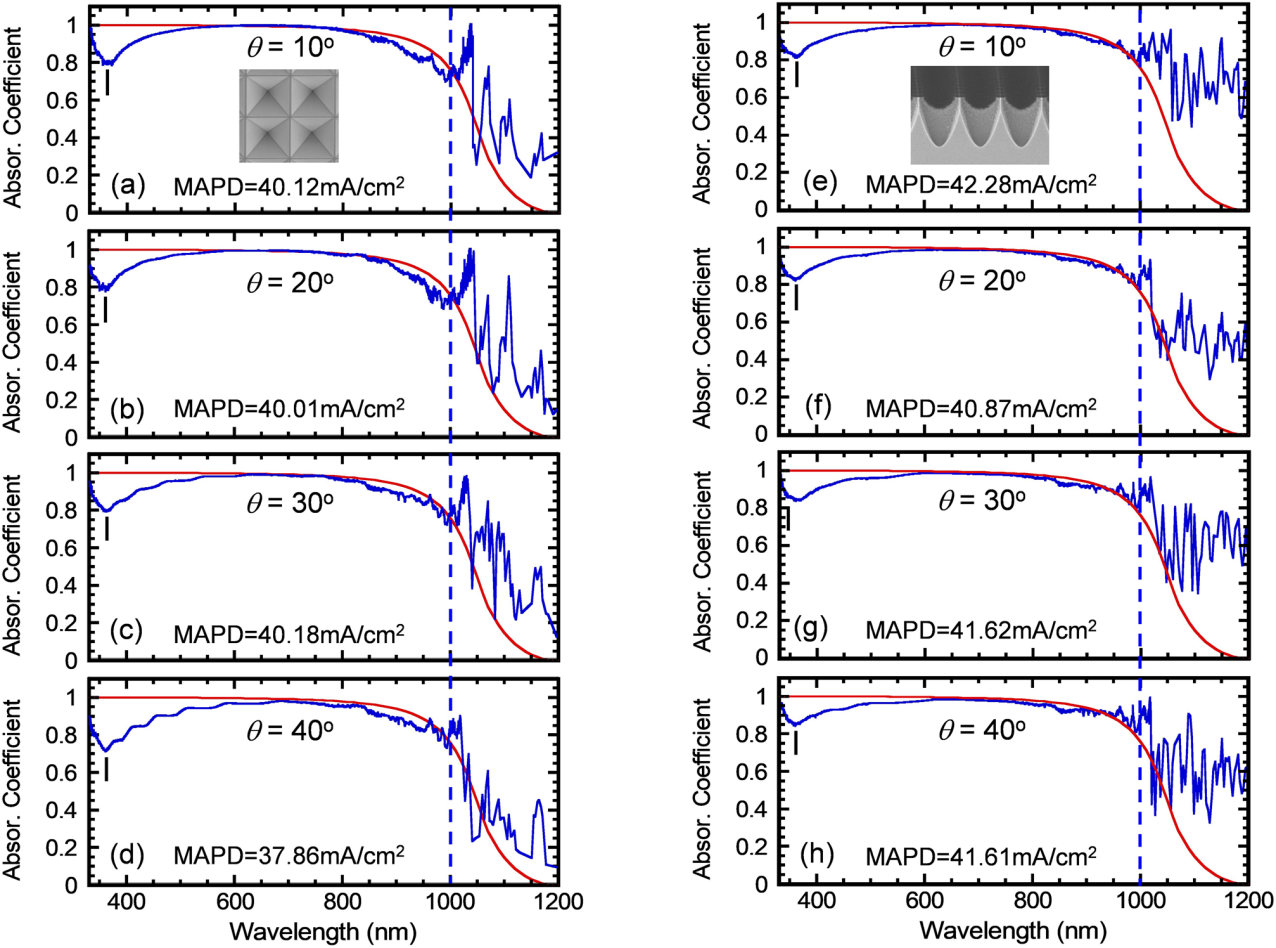
Angular dependence of absorption spectra over λ = 330–1,200 nm. (**a**)**–**(**d**) shows the measured absorption spectra of the IP cell structure for light incident at θ = 10°, 20°, 30° and 40°, respectively. (***e***)**–**(***h***) shows the measured absorption spectra of the Teepee cell structure for light incident at θ = 10°, 20°, 30° and 40°, respectively.

In the following, we investigate the angular response of the Inverted Pyramid and Teepee PhC structures from θ = 10° to 60°. Figure 8a–d shows the measured absorption spectra of the Inverted Pyramid PhC for light incident at θ = 10°, 20°, 30° and 40°, respectively. For the tilt angle testing, there is no absorption data presented here for λ = 300–330 nm range as the signal is too weak and beyond the detection limit of our Ocean Optics spectrometer. The weak signal is due to combined effects of a reduced beam size (~ 5 mm) and a weak light source intensity in this spectral range. Nonetheless, we found the sample's UV absorption at λ = 375 nm remains high at ~ 80% for θ = 10–30° and then drops slightly to ~ 70% for θ = 40°. Additionally, the sample's visible absorption over λ = 550–800 nm range is near the Lambertian limit for θ = 10°–30° and drops slightly below the limit for θ = 40°. Finally, the sample's near infrared (1,000–1,200 nm) absorption far exceeds the Lambertian limit for θ = 10°–30°, but only slightly exceeds the limit for θ = 40°. Therefore, the Inverted Pyramid PhC maintains its excellent absorption performance for θ = 10°–30°, but not nearly as well for θ = 40°. Similarly, Fig. 8e–h shows the measured absorption spectra of the Teepee PhC for light incident at θ = 10°, 20°, 30° and 40°, respectively. For θ = 10–40°, the Teepee PhC maintains a high absorption in the UV, visible and near-infrared. Its UV absorption at λ = 375 nm is maintained at ~ 83% for all angles studied, and its near-infrared absorption at λ = 1,000–1,200 nm is 50–80% for all angles studied, which far exceeds the Lambertian limit. We believe the reduced angular dependence of optical absorption of the Teepee PhC is due to its more gradual optical index profiles^18,27–29^. This Gaussian index profile can lead to an excellent optical antireflection that is broadband (λ = 300–1,200 nm) and almost angular independent (θ = 0°–60°).

Table 1 shows a summary of the angular dependence of MAPD over 330–1,200 nm range under AM1.5G illumination for the 10-μm thick silicon Inverted Pyramid and Teepee PhC structures. As a reference, MAPD over 330–1,200 nm range corresponding to Lambertian limit is 39.51 mA/cm^2^. We experimentally demonstrated the MAPD for the Inverted Pyramid PhC exceeds the Lambertian limit at θ = 10°, 20°, and 30°, although it falls below the Lambertian limit at larger light incident angles from θ = 40°-60°. Moreover, we found experimentally that the MAPD for the Teepee PhC exceeds the Lambertian limit by 2.77, 1.36, 2.11, 2.10, 1.80 and 1.48 mA/cm^2^ for θ = 10°, 20°, 30°, 40°, 50° and 60°, respectively. This excellent MAPD performance is due to an efficient coupling of plane-waves to the long-lifetime PIR modes of the PhC utilizing all-θ, all-λ graded-index antireflection design.

**Table 1:** A summary of the angular dependence of MAPD for 330–1,200 nm range under AM1.5G illumination for both the 10-μm thick silicon Inverted Pyramid and Teepee PhC cell structures. The MAPD corresponding to the Lambertian limit for λ = 330–1,200 nm range is 39.51 mA/cm^2^.

Samples	Incident angle					
	10 deg	20 deg	30 deg	40 deg	50 deg	60 deg
Inverted pyramid photonic-crystal (mA/cm^2^)	40.12	40.01	40.18	37.86	36.25	35.57
Teepee photonic-crystal (mA/cm^2^)	42.28	40.87	41.62	41.61	41.33	40.99

## Summary

In summary, we proposed and realized a new light-trapping structure for thin film silicon solar cells based on wave-interference optics. This is a singular experimental demonstration of above-Lambertian solar absorption integrated over the entire wavelength range of 300–1,200 nm. We constructed two types of 10*-*μm thick silicon simple-cubic PhC cell structure: the Inverted Pyramid PhC and the Teepee PhC. In contrast to the Lambertian cell and planar cell structures, higher solar absorption in the 950–1,200 nm spectral range due to multiple resonant absorption peaks was demonstrated. These peaks in the absorption spectra originate solely from wave-interference effects that are absent in Lambertian light-trapping. Furthermore, we found the maximum-achievable-photocurrent-density (MAPD) under AM1.5G illumination at a 4-degree incident angle to be 41.29 and 41.52 mA/cm^2^ for the Inverted Pyramid and Teepee PhC, respectively. These values exceed the MAPD (= 39.63 mA/cm^2^) corresponding to the Lambertian limit for a 10-μm thick silicon despite the architectures not being fully optimized for light-trapping. In the experimentally measured systems, we observed overall absorption up to 2 mA/cm^2^ beyond the Lambertian limit. Numerical simulations of Maxwell’s equations in fully optimized, 10-μm thick silicon, photonic crystal structures^1^ suggest that the overall absorption would be up to 4 mA/cm^2^ beyond the Lambertian limit. The agreement of present measurements with theory lends credence to this more far-reaching prediction and the resulting possibility of thin-silicon solar cells with efficiencies surpassing 30%.
